# Calcium Silicate-Based Versus Conventional Sealers in Nonsurgical Endodontic Retreatment: A Systematic Review and Meta-Analysis

**DOI:** 10.7759/cureus.117870

**Published:** 2026-10-13

**Authors:** Omar Alshammari, Bayan A Rashed, Fatimah M Alghomgham, Shaheen W Al Hajaj, Turki W Almuhayfith, Aya A Hejazi, Souhip Al-Harbi, Khalid A Alghamdi, Mashael Mujalli, Mohammed A Alshehri, Khalid M Alzahrani, Maha A Alatawi, Latifah A Al-Ali, Abdulaziz Alribi, Ali S Metwaly

**Affiliations:** 1 General Dentistry, Al-Farwaniya Specialty Dental Center, Farwaniya, KWT; 2 Endodontics, Airbase Hospital, Dhahran, SAU; 3 General Dentistry, Dr. Sukainah Al Sulaiman Dental Clinic, Qatif, SAU; 4 Endodontics, Istanbul University, Istanbul, TUR; 5 Endodontics, King Faisal University, Al-Ahsa, SAU; 6 General Dentistry, Umm al-Qura University, Jeddah, SAU; 7 General Dentistry, Batterjee Medical College, Jeddah, SAU; 8 General Dentistry, Riyadh Elm University, Riyadh, SAU; 9 General Dentistry, Jazan Health Cluster, Jazan, SAU; 10 General Dentistry, King Khalid University, Abha, SAU; 11 Endodontics, Al Baha University, Al-Baha, SAU; 12 General Dentistry, University of Ha'il, Tabuk, SAU; 13 General Dentistry, Kuwait Ministry of Health, Al Jahra, KWT; 14 Endodontics, King Saud bin Abdulaziz University for Health Sciences, Riyadh, SAU; 15 Precision Medicine, Faculty of Pharmacy, Alexandria University, Alexandria, EGY

**Keywords:** bioceramic sealer, calcium silicate, endodontic retreatment, meta-analysis, periapical healing, postoperative pain, root canal sealer

## Abstract

This systematic review and meta-analysis aimed to determine whether obturation with a calcium silicate-based sealer alters periapical healing, postoperative pain, analgesic consumption, apical sealer extrusion, or tooth survival compared with a conventional sealer in permanent teeth undergoing orthograde nonsurgical retreatment; to establish whether any effect on healing is stable across loose and strict success criteria; and to determine whether the published evidence permits a causal statement about sealer chemistry independent of obturation technique.

Seven databases, two trial registries, and two grey literature sources were searched from inception to August 2026, with citation chasing and hand-searching. Randomized and non-randomized comparative studies of orthograde nonsurgical retreatment comparing a calcium silicate-based sealer with any non-calcium-silicate sealer were eligible. Loose (healed plus healing) and strict (complete resolution) radiographic success at ≥ 12 months were co-primary. The designs were synthesized separately. In two studies, pooling was conditional on a prespecified heterogeneity screen (I² < 50% and p_Q_ ≥ 0.10); outcomes failing the screen were reported under Synthesis Without Meta-analysis (SWiM). Risk of bias was assessed with Revised Cochrane Risk-of-Bias tool for randomized trials (RoB 2) and Risk of Bias in Non-randomized Studies - of Interventions (ROBINS-I), and certainty with Grading of Recommendations, Assessment, Development and Evaluation (GRADE). Nine studies were included; five contributed data (276 teeth). Under loose criteria, the two randomized trials disagreed (risk ratio (RR) 1.14, 95% confidence interval (CI) 0.90-1.43 and 0.93, 0.82-1.07; I² = 51.4%) and were not pooled. Under strict criteria, both favored the conventional sealer and were consistent (I² = 0%), giving a pooled RR of 0.56 (0.27-1.15; 67 teeth), equivalent to 189 fewer complete resolutions per 1,000 teeth (310 fewer to 62 more). The exploratory ratio of RRs between criteria was 1.87 (0.89-3.90), an interval that includes 1. Postoperative pain did not differ at 24 hours (0.93, 0.53-1.64) or 48 hours (0.85, 0.43-1.67; 165 teeth). No sealer extrusion occurred in either arm of the only trial reporting it. No retreatment-specific survival data exist. The prespecified subgroup analysis of obturation technique could not be performed: no study contributing healing data matched the technique between arms. Available evidence represented 35% of the optimal information size for strict healing, 4% for loose healing and 0.8% for pain at 24 hours. Certainty was very low for all healing outcomes and low for pain.

In nonsurgical retreatment, the direction of the estimated effect of sealer chemistry differed between definitions of success: no difference under loose criteria, and a point estimate favoring conventional sealers under strict criteria that the data are too imprecise to confirm or exclude. Because the ratio of RRs includes 1, this difference is exploratory and does not establish that the effect depends on the criterion. Because sealer chemistry is confounded with obturation technique in every study reporting healing, the contrast measured is between obturation strategies rather than between sealers. The field is far from an answer, and a trial of approximately 192 teeth using strict criteria would be decisive.

## Introduction and background

Post-treatment apical periodontitis is the most common reason for a root-filled tooth to return to care, and orthograde nonsurgical retreatment remains the first-line response [1,2]. Retreatment is not a primary treatment repeated. Retreatment differs from primary treatment in several aspects: the existing root filling must be removed before disinfection can begin, the remaining microorganisms form a mature biofilm that has survived a previous course of treatment, the canal walls have already been instrumented, and the periapical lesion is usually older and larger at baseline [1,2]. Pooled estimates of retreatment success are lower and more dispersed, and the prognostic weight of individual technical variables differs between the two settings [1,2]. Therefore, evidence in primary treatment transfers to retreatment only by assumption.

Hydraulic calcium silicate-based sealers were introduced on a biological rationale that is most relevant precisely where the microbiological and healing challenges are greatest. They set in the presence of tissue fluid, release calcium hydroxide, and raise the local pH sufficiently to exert antibacterial activity, nucleate apatite at the dentine interface, and show lower cytotoxicity than epoxy resin sealers in cell culture [3-5]. Because they are hydraulic rather than thermoplastic, they are delivered by a single-cone or sealer-based technique rather than by warm vertical compaction, and this coupling of material chemistry to obturation technique is a structural feature of the clinical literature rather than an incidental one [6].

The evidence regarding these materials is concentrated almost entirely on primary treatment and early postoperative pain. Systematic reviews of radiographic outcomes [7-12], at least five meta-analyses of postoperative pain [9,13-16], a network meta-analysis of sealer type and pain [17], and two umbrella reviews [18,19] converge on the same conclusion: differences between sealer classes are small, short-lived, and of doubtful clinical importance. These syntheses share a mixed population. Retreatment cases, where eligible, enter the pooled estimate alongside primary cases and are outnumbered by them; therefore, the retreatment signal is diluted rather than measured. The umbrella reviews identify the under-representation of retreatment as a limitation of the trials they summarize [18,19].

One systematic review addressed the question, comparing calcium silicate with resin-based sealers for periapical healing after retreatment, but it performed no quantitative synthesis and was restricted to randomized evidence [20]. The gap it leaves is fourfold. First, no review has pooled comparative outcome data restricted to nonsurgical retreatment. Second, no review has brought the comparative observational evidence, which contains the only cohort recruited from retreatment cases [21], alongside the randomized evidence under design-appropriate appraisal and separate synthesis. Third, no review has addressed the confounding of sealer chemistry with obturation technique: in most eligible studies, the calcium silicate arm is obturated by single cone or sealer-based delivery, while the comparator receives warm vertical compaction or cold lateral condensation, and in at least one cohort, the correspondence is perfect, every resin case being compacted and every bioceramic case single-coned [22]. The contrast estimated is between two obturation strategies, not between two sealers.

Fourth, no review has asked whether the answer is stable across the two radiographic definitions of success that endodontic outcome research uses. Loose criteria count complete and partial resolution together, whereas strict criteria count complete resolution only. If a material alters the rate at which a lesion resolves without altering whether it resolves, the two definitions will not agree at 12 months, and a literature that reports whichever definition is more favorable will appear more consistent than it is. This concern is not new to endodontic outcome research [23], but it has never been examined for sealer chemistry.

Two developments make this question answerable now. The first randomized trial confined to retreatment and assessed volumetrically on cone-beam computed tomography has been published [24], and a comparative cohort restricted to retreated teeth with periapical lesions is now available [21]. Recent single-arm cohorts of calcium silicate-based retreatment, including the first with three-dimensional outcome assessment, report success at the upper end of the retreatment literature but cannot separate the sealer from the protocol surrounding it [25-27].

Therefore, the objectives of this review were: (i) to determine whether obturation with a calcium silicate-based sealer alters radiographic periapical healing, postoperative pain, analgesic consumption, apical sealer extrusion, or tooth survival after orthograde nonsurgical retreatment; (ii) to establish whether any effect on healing is stable across loose and strict success criteria, treating both as co-primary; (iii) to synthesize randomized and non-randomized evidence separately and report discordance between designs as a finding rather than averaging it away; and (iv) to determine whether the available studies permit any causal statement about sealer chemistry independent of the obturation technique with which it is confounded.

## Review

Methods

Reporting Standards

Reporting follows Preferred Reporting Items for Systematic Reviews and Meta-analyses (PRISMA) 2020 [28], PRISMA-S for the search [29], Synthesis Without Meta-analysis (SWiM) for outcomes not amenable to meta-analysis [30], and Preferred Reporting Items for RAndomized Trials in Endodontics (PRIRATE) 2020 where applicable [31].

Eligibility Criteria

Study eligibility was formulated using the population, intervention, comparison, outcomes, and study design (PICOS) framework [32]. The population comprised patients of any age with permanent teeth that had previously been root-filled and required orthograde nonsurgical retreatment for post-treatment apical periodontitis, whether persistent or secondary, diagnosed clinically and/or radiographically. Primary root canal treatment, surgical retreatment, apexification, and regenerative procedures were not eligible for inclusion. Obturation using a hydraulic calcium silicate-based (bioceramic) sealer of any brand, presentation, or delivery method was compared with obturation using a non-calcium-silicate sealer: epoxy resin-based, zinc oxide-eugenol-based, calcium hydroxide-based, silicone-based, or glass-ionomer-based.

The primary outcome was radiographic periapical healing at a minimum of 12 months, extracted under two prespecified co-primary definitions: loose (healed plus healing, that is, complete or partial resolution with absence of symptoms) and strict (complete resolution only), scored with the Periapical Index [33], its cone-beam equivalent [34], the Friedman and Mor criteria [35], or a documented equivalent. Neither definition was a sensitivity analysis of the other, and the analytical rules below were applied to both. The secondary outcomes were incidence of postoperative pain at each reported time point to seven days; pain intensity on a visual analogue scale at 24 and 48 hours; rescue analgesic consumption; apical sealer extrusion; and tooth survival or extraction at the longest follow-up. Furthermore, a minimum arm size of 10 teeth per group was required for studies to be included in the quantitative synthesis.

Randomized controlled trials (RCTs) of any allocation structure and non-randomized comparative clinical studies were eligible; a comparator group obturated with a non-calcium-silicate sealer was mandatory. Single-arm studies, uncontrolled case series, laboratory and animal studies, case reports, narrative reviews, and abstracts without extractable data were excluded. Studies enrolling both primary and retreatment cases were eligible only where retreatment-specific outcome data were recoverable from the published report or its supplementary material. No language or date restrictions were applied.

Information Sources, Search and Study Selection

MEDLINE via PubMed, Embase, CENTRAL, Scopus, Web of Science Core Collection, LILACS, and Google Scholar were searched from inception to August 2026. ClinicalTrials.gov and the WHO International Clinical Trials Registry Platform were searched for trials, and OpenGrey and ProQuest Dissertations were searched for grey literature. Backward and forward citation chasing was performed on every included study and on each existing systematic review in the field [7-12,20], and the last five volumes of three specialty journals were hand-searched. The search combined three concept blocks - retreatment; calcium silicate or bioceramic sealers; and comparators and clinical outcomes - using controlled vocabulary and free text adapted to each interface. Records were deduplicated and screened in Rayyan [36] by two reviewers independently at title, abstract, and full text, with a third resolving disagreements; agreement at full text was quantified as Cohen's kappa and interpreted per Landis and Koch [37].

Data Extraction and Unit-of-Analysis Considerations

A piloted form was completed independently and in duplicate for all outcome data, recording study identifiers and design; country, setting, and operator experience; number of patients enrolled and analyzed per arm; number of retreatment teeth per arm where the population was mixed; baseline diagnosis, Periapical Index distribution, and lesion size; sealer brand and comparator class; the obturation technique used in each arm separately; instrumentation, irrigation, and activation protocol; number of visits and intracanal medication; imaging modality and outcome index; follow-up duration and recall rate; attrition and postrandomization exclusions; and all outcome data as reported. Where a study reported outcomes only as pain-severity category counts, pain incidence was derived as the complement of the no-pain category and checked against reported arm totals at every time point.

Where several teeth from one patient were included, the tooth was the unit of analysis, and the resulting clustering was acknowledged as a limitation. For split-mouth trials, within-patient paired estimates were used where reported; where only marginal counts were available, the trial was analyzed as parallel, which is conservative, and the effect of assuming intracluster correlations of 0.2, 0.4, and 0.6 was examined in a sensitivity analysis [38]. Multi-arm trials contributed each eligible arm once against the shared intervention arm, with the intervention arm divided across comparisons to prevent double counting. Multiple reports of one cohort were treated as one study, the longest follow-up supplying the primary outcome.

Risk of Bias

Randomized studies were appraised using Revised Cochrane Risk-of-Bias tool for randomized trials (RoB 2), using the split-mouth variant where applicable [39]; non-randomized studies with Risk of Bias in Non-randomized Studies - of Interventions (ROBINS-I) against a prespecified confounder list comprising baseline lesion size, tooth type, pulpal and periapical diagnosis, number of visits, obturation technique, operator, coronal restoration quality, and follow-up duration [40]. Judgements were made independently and in duplicate and resolved by discussion, with the supporting text from each source article recorded domain by domain. Traffic-light and weighted summary plots were produced separately by design in Python 3 using Matplotlib [41]. No composite quality score was computed, and risk-of-bias judgements were not used to weight studies. Studies rated at critical risk of bias on ROBINS-I were excluded from all syntheses.

Synthesis

Randomized and non-randomized evidence were pooled separately and never combined; the two were compared narratively, and discordance between them was reported as a finding. Dichotomous outcomes were summarized as risk ratios (RRs) with 95% CIs and continuous outcomes as mean differences (MD), with standardized mean differences (SMD) (Hedges' g) used only where scales differed.

With three or more studies, a random-effects (RE) model was used with restricted maximum likelihood estimation of τ², the Hartung-Knapp-Sidik-Jonkman adjustment to the confidence interval [42], and a 95% prediction interval. With two studies, pooling was permitted only if a prespecified heterogeneity screen was passed: at two studies, Cochran's Q has minimal power, and I² has a confidence interval spanning almost its entire range, so neither can confirm homogeneity, and both were therefore used only in the conservative direction. Pooling required both I² < 50% and pQ ≥ 0.10; if the screen was passed, a Mantel-Haenszel common-effect estimate [32] without continuity correction was reported as primary, accompanied by an explicit statement that between-study variance is not estimable, with an RE estimate reported only as a sensitivity analysis. If the screen was failed, no pooled estimate was produced, and the outcome was reported under SWiM [30]. With one study, no pooling was performed. For every outcome not pooled, a structured report was produced giving each study's estimate and interval, the direction of effect, whether the interval excluded the null, a vote count of directions and the range of individual effects; forest plots for non-pooled outcomes display individual estimates only, with no summary diamond and no heterogeneity footer [43]. For transparency, the estimate the screen withheld was computed and reported, labelled as not being used for inference.

Where events were rare, Mantel-Haenszel without continuity correction was preferred to inverse-variance weighting, and the Peto odds ratio [32] was computed as a check. Outcomes with no events in either arm of every contributing study were not forced onto a ratio scale; they were reported with the exact one-sided 97.5% upper bound on the underlying risk in each arm. Because two contributing arms reached 100% success, the primary outcome was additionally re-parameterized on the failure scale.

When the loose and strict estimates differed in direction, the difference was quantified post hoc as a ratio of RRs computed on the log scale, as the difference between the two log RRs [44]. The two estimates derive from overlapping patients and are positively correlated; therefore, treating them as independent overstates the variance, and the resulting interval is conservative; the analysis is presented descriptively rather than as a hypothesis test.

Prespecified subgroup analyses, each conditional on at least four studies per level, were: obturation technique matched between arms versus not matched, as the primary co-intervention analysis; comparator class; follow-up duration; imaging modality; and baseline lesion size. Before any estimate was computed, the availability of each level was tabulated; where a level contained no contributing study, the analysis was reported as structurally unavailable rather than as underpowered, because the distinction matters for what future research must do. Where design and another variable are themselves confounded across the available studies, no attempt is made to separate them statistically.

he following sensitivity analyses were prespecified: (i) leave-one-out omission of each study; (ii) exclusion of studies at high or critical risk of bias; (iii) relaxation of the minimum arm-size criterion; (iv) use of alternative estimators of between-study variance (τ²) [45]; (v) variation of the intracluster correlation assumed for split-mouth trials; (vi) best-case and worst-case imputation for outcome events whose allocation to an arm was not reported; and (vii) alternative codings of any study containing an unresolved internal numerical inconsistency.

For each outcome, the optimal information size was computed as the total sample size a single adequately powered trial would require to detect the observed effect at two-sided α = 0.05 and 80% power, and the available evidence expressed as a percentage of it [46]. For each contributing study, a fragility index, or, for non-significant results, a reverse fragility index was computed as the smallest number of outcome events whose reassignment would change the significance of the result [47]. Contour-enhanced funnel plots and Egger's test [48] were prespecified for any analysis containing at least ten studies; where that threshold was not reached, no plot or test was produced and the inability to assess small-study effects is reported as a limitation rather than substituted by an underpowered test.

Certainty of the Evidence and Absolute Effects

Certainty was rated with Grading of Recommendations, Assessment, Development and Evaluation (GRADE) for each outcome and each design stratum separately [49,50]. Randomized evidence started at high certainty and non-randomized evidence at low. Indirectness was downgraded wherever sealer chemistry and obturation technique differed between arms, because the contrast then does not isolate the intervention of interest. Inconsistency was downgraded wherever the heterogeneity screen failed. Imprecision was rated against the optimal information size and the position of the confidence limits relative to clinical decision thresholds [51]. Absolute effects were computed from the assumed comparator risk and the pooled relative effect and reported per 1000 teeth, constrained to the interval 0-1000, and where the relative effect is so imprecise that the absolute estimate carries no information, the cell states this instead of printing a number.

Software

Analyses were performed in R version 4.6.1 (R Foundation for Statistical Computing, Austria) using the meta [52] and metafor [53] packages, following the Cochrane Handbook version 6.5 [32]. Risk-of-bias and certainty figures were produced in Python 3 with Matplotlib [41].

Results

Study Selection and Characteristics

Nine studies in ten reports met the eligibility criteria (Figure 1). Five studies contributed quantitative data, comprising 276 teeth: two randomized trials of periapical healing (67 teeth) [24,54], two randomized trials of postoperative pain (165 teeth) [55,56], and one retrospective cohort of periapical healing (44 teeth) [21]. Three studies (reported across four publications) met the initial population and intervention criteria but were excluded from the synthesis because retreatment-specific results were not recoverable from the published reports [57-60], thus failing this specific eligibility criterion. One retrospective cohort was excluded from all syntheses [22].

**Figure 1:**
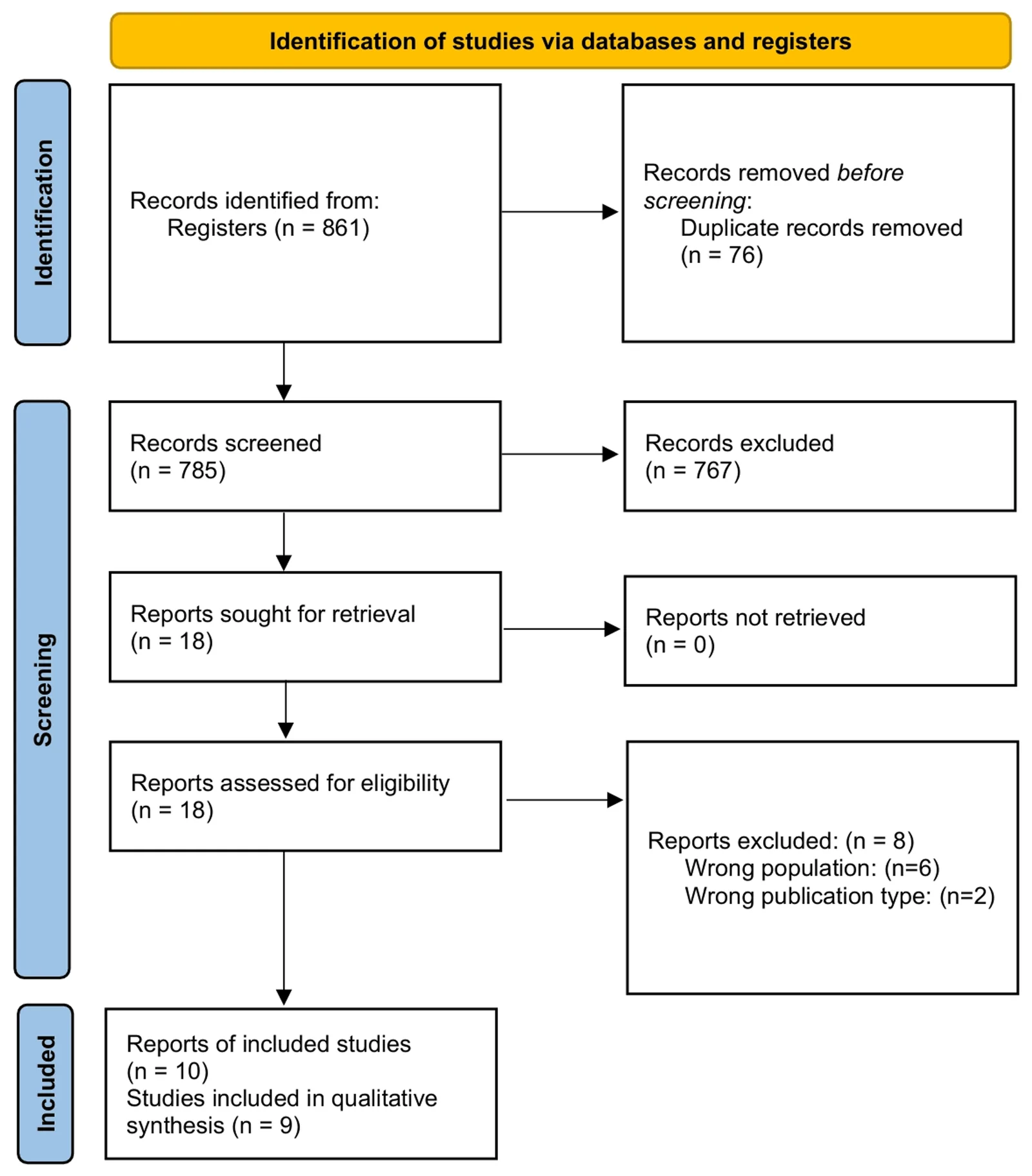
PRISMA 2020 flow diagram PRISMA: Preferred Reporting Items for Systematic Reviews and Meta-analyses

Characteristics are given in Table 1. Studies were conducted in Lithuania, Croatia, Syria, Türkiye, Egypt, Italy, Thailand, and Qatar and published between 2018 and 2025. Five studies enrolled only retreatment cases [21,24,54,55,60], and four enrolled mixed populations [22,56-59], wherein [57,58] are two reports of the same trial. Of these, two reported an extractable retreatment stratum [22,56]. The calcium silicate sealers used were BioRoot RCS, TotalFill BC, Ceraseal, iRoot SP, and Sure Seal Root; the comparators were AH Plus in seven studies, a zinc oxide-eugenol sealer in one, and a calcium hydroxide sealer in one three-arm trial. Two studies used cone-beam computed tomography for outcome assessment [24,56], and the remainder used periapical radiography. Bardini et al. 2021 [57] and Bardini et al. 2025 [58] represent separate reports of 12-month and 48-month follow-ups, respectively, originating from the same underlying clinical trial.

**Table 1:** Characteristics of the nine included studies Kamacı Esen and Kalabalık [21]: Mean follow-up 11.9 months (CSS) versus 23.6 months (comparator); range not reported. CSS: Calcium silicate-based sealer; CBCT: Cone-beam computed tomography; ZOE: Zinc oxide-eugenol

Study, design	Country, population	Retreated teeth analyzed, CSS/comparator (patients randomized or enrolled)	Outcomes contributed	CSS arm (sealer, technique)	Comparator arm (sealer, technique)	Imaging	Follow-up
Graunaite et al. [55], RCT (split-mouth)	Lithuania; retreatment only	57 / 57	Pain, extrusion	TotalFill BC; warm vertical	AH Plus (epoxy); warm vertical	Periapical	7 days
Düzgün et al. [54], RCT (3-arm)	Türkiye; retreatment only	Randomized 32 / 32 / 32; analyzed 26 / 25 (epoxy); 29 (CaOH)	Pain, analgesic	Sure Seal Root; lateral compaction	AH Plus; Sealapex; lateral compaction	Periapical	7 days
Pažin 2025 [24], RCT	Croatia; retreatment only	19 / 18 (50 patients randomized, 48 evaluated at 1 year; arm sizes derived from reported percentages)	Healing (loose, strict)	BioRoot RCS; single cone	AH Plus; cold lateral	CBCT	12 months
Wahbi et al. [56], RCT	Syria; mixed (untreated + retreatment)	15 / 15 (retreatment stratum of 60 teeth in 41 patients)	Healing (loose, strict)	TotalFill BC; single cone	AH Plus; cold lateral	CBCT	12 months
Kamacı Esen and Kalabalık [21], retrospective cohort	Türkiye; retreatment only	28 / 16 (44 teeth in 44 patients)	Healing (loose, strict), ΔPAI	Ceraseal; single cone or lateral	AH Plus; single cone or lateral	Periapical	11.9 / 23.6 months
Bani-Younes et al. [22], retrospective cohort	Qatar; mixed	5 / 21	None (excluded)	TotalFill BC; single cone	AD Seal (resin); warm vertical	Periapical	≥ 12 months
Bardini at al. [57,58], RCT	Italy; mixed (primary + secondary)	26 / 10; 9 / 8	None (awaiting classification)	BioRoot RCS; single cone	Pulp Canal Sealer EWT (ZOE); warm vertical	Periapical	12 and 48 months
Kangseng et al. [59], retrospective matched cohort	Thailand; mixed	20 / 20	None (awaiting classification)	BioRoot RCS, iRoot SP, Ceraseal; cold hydraulic condensation	AH Plus; warm vertical	Periapical	Median 15 months
Ghobashy and Fakhr [60], RCT	Egypt; retreatment only	50 / 50	None (awaiting classification)	Bioceramic (brand not reported); single cone	Epoxy resin; cold lateral	Periapical	7 days

Risk of Bias

Of the six randomized studies, five were assessed for risk of bias (Figures 2,3), as one study [60] did not contribute extractable data. Of these five, three were judged to have some concerns and two at high risk of bias. Both high ratings arose from features bearing on this review. In one trial, 96 patients were randomized (32 per arm), and 16 were excluded after randomization: 10 for loss to follow-up and 6 because sealer extrusion had occurred. This left 80 analyzed (calcium silicate 26, epoxy resin 25, calcium hydroxide 29), corresponding to 6, 7 and 3 exclusions per arm, respectively [54]. The allocation of the six extrusion exclusions was not reported. This is a departure from the intention-to-treat principle with a plausible mechanism linking exclusion to the outcome, and it removes extrusion as an analyzable randomized endpoint in that trial. Allocation followed a coin flip for the first patient of each day and deterministic alternation thereafter, making the sequence foreseeable; the resulting baseline imbalance fell on the variable defining the population, with 26 of 39 secondary treatments in the bioceramic group against 10 of 30 in the comparator (p = 0.0060) [57]. Allocation concealment was not described in any of the five trials, and in three the obturation technique is visually distinguishable on the radiograph or scan being scored, so complete masking of outcome assessment was not achievable even where attempted.

**Figure 2:**
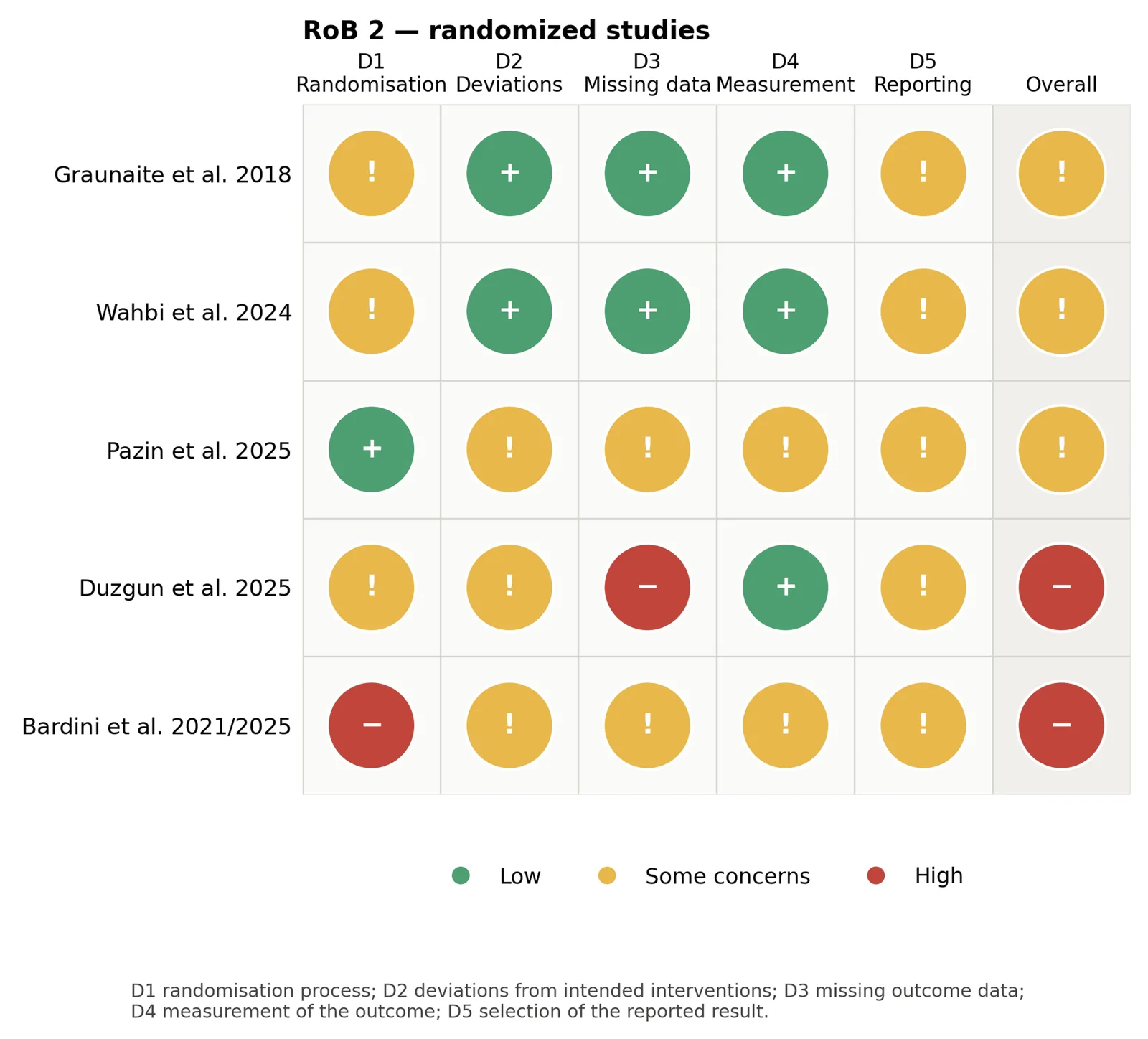
Risk of bias in the five randomized studies (RoB 2), by domain and overall Green plus: Low risk; Amber exclamation: Some concerns; Red minus: High risk [24,54-58] RoB 2: Revised Cochrane Risk-of-Bias tool for randomized trials

**Figure 3:**
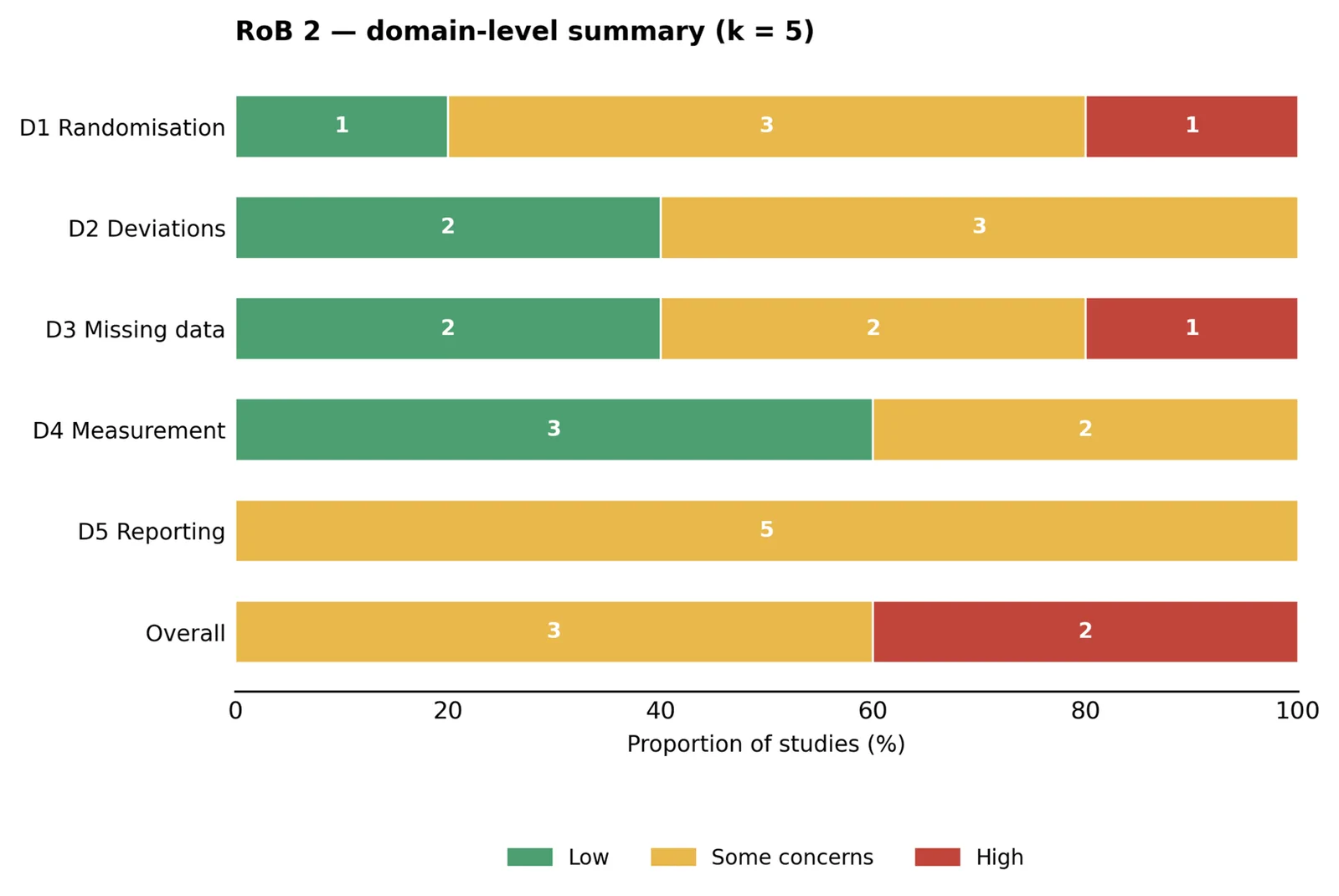
Domain-level distribution of RoB 2 judgements across the five randomized studies RoB 2: Revised Cochrane Risk-of-Bias tool for randomized trials

Of the three non-randomized studies (Figures 4, 5), one was rated as moderate risk [59], one as serious risk [21], and one as critical risk [22]. In the serious-risk cohort, baseline Periapical Index 5 was present in 12 of 28 teeth in the calcium silicate arm against 1 of 16 in the resin arm (p = 0.03), and mean follow-up differed (11.9 versus 23.6 months), with no adjustment; the same clinician selected the sealer, delivered the treatment, and scored the radiographic outcome without masking. The critical-risk cohort was rated so because the sealer type was confounded with the obturation technique; every resin case was obturated by warm vertical compaction and every bioceramic case by single cone, leaving no residual variation from which a sealer effect could be separated by any analytic method [22]. Therefore, that study was excluded from all syntheses; it failed the minimum arm-size rule, its bioceramic retreatment arm containing five teeth, and its retreatment stratum was double-zero for failure; so the exclusion is triply determined and the quantitative results are unaffected.

**Figure 4:**
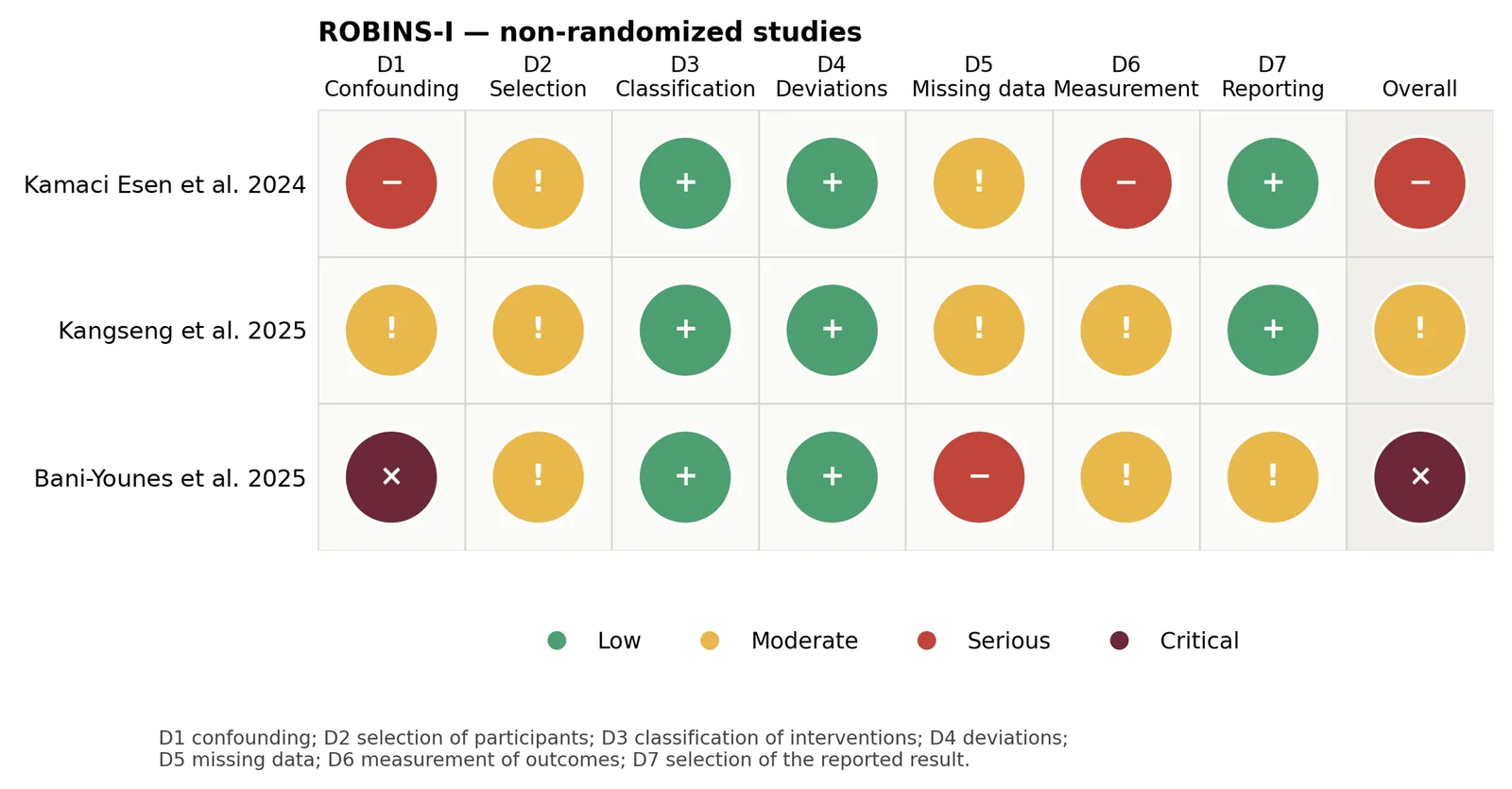
Risk of bias in the three non-randomized studies (ROBINS-I), by domain and overall [21,22,59] ROBINS-I: Risk of Bias in Non-randomized Studies - of Interventions

**Figure 5:**
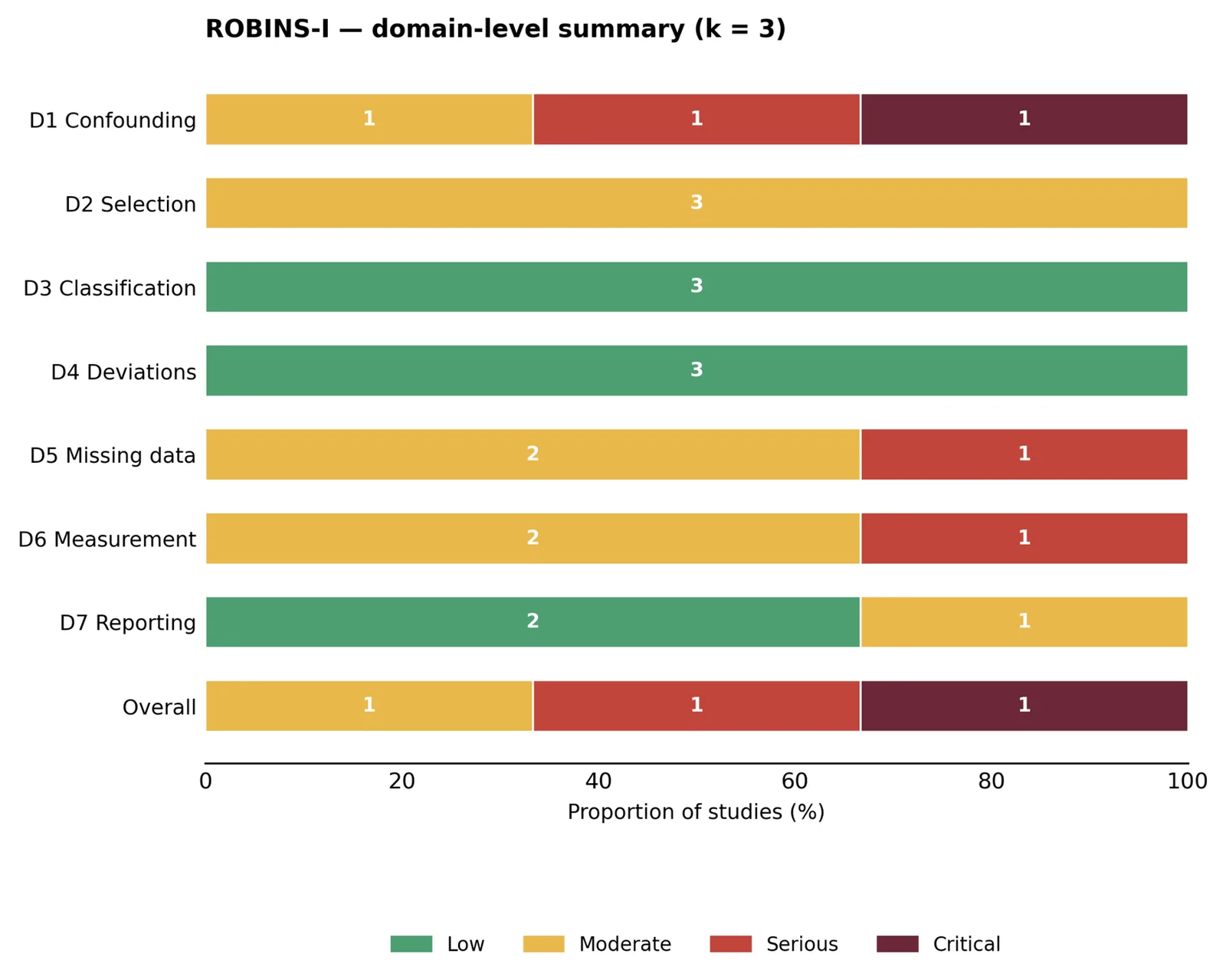
Domain-level distribution of ROBINS-I judgements across the three non-randomized studies ROBINS-I: Risk of Bias in Non-randomized Studies - of Interventions

Primary Outcome: Periapical Healing under Loose Criteria

The two randomized trials disagreed in direction. One reported 18 of 19 successes with the calcium silicate sealer against 15 of 18 with the epoxy resin comparator (RR 1.14, 95% CI 0.90-1.43) [24], while the other reported 14 of 15 against 15 of 15 (RR 0.93, 0.82-1.07) [56]. Heterogeneity reached I² = 51.4% with pQ = 0.152; therefore, the prespecified screen was not passed, and no pooled estimate was produced (Figure 6). Neither interval excluded the null, and the range of individual effects was 0.93 to 1.14. For transparency, the estimate the screen withheld was a common-effect RR of 1.04 (0.90-1.19); it assumes a common effect the data do not support and is not used for inference.

**Figure 6:**
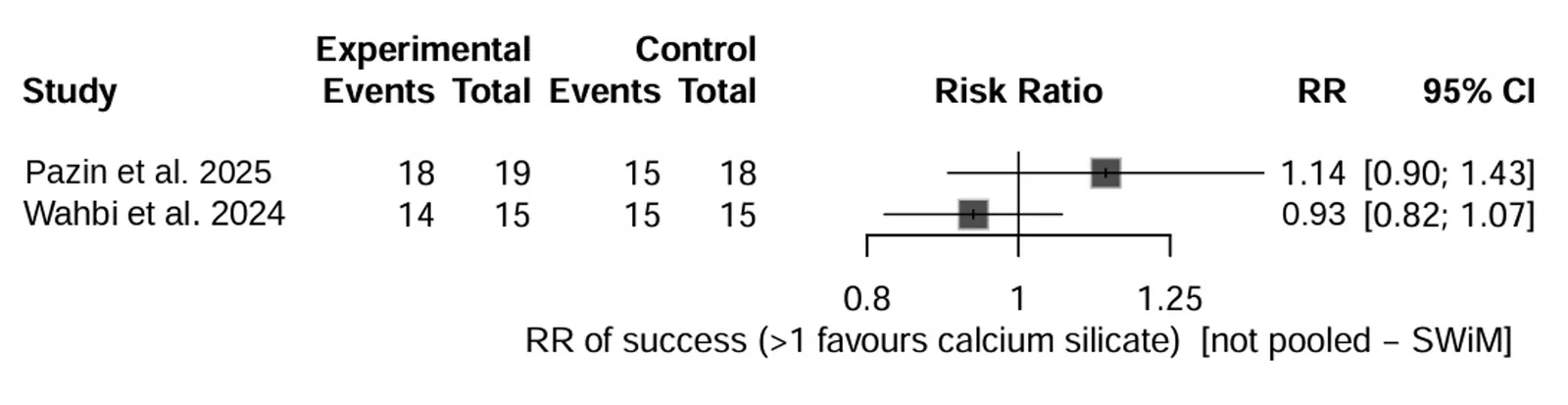
Periapical healing under loose criteria, randomized stratum Individual study estimates are shown without a summary diamond because the prespecified heterogeneity screen was not passed (I² = 51.4%, pQ = 0.152) and no pooled estimate was produced [24,56]. RR: Risk ratio; CI: Confidence interval; SWiM: Synthesis Without Meta-analysis

In the non-randomized stratum, a single eligible cohort reported 28 of 28 successes with the calcium silicate sealer against 15 of 16 with the epoxy resin comparator (RR 1.07, 0.94-1.21) [21]. This cohort reported follow-up only as arm means (11.9 months in the calcium silicate arm and 23.6 months in the comparator arm), without a range or minimum, so it cannot be confirmed that every tooth met the 12-month eligibility threshold, and the calcium silicate mean implies that some did not. The cohort was retained as the only comparative non-randomized evidence. It is reported separately, contributes to no randomized estimate, and was downgraded for indirectness. Because shorter follow-up allows less time for healing, this deviation would be expected to bias the comparison against the calcium silicate arm. With the minimum arm-size and critical-risk rules both relaxed, adding the excluded cohort gave 1.05 (0.96-1.15), confirming that the exclusion does not drive the result.

Primary Outcome: Periapical Healing under Strict Criteria

Under strict criteria, the same two randomized trials agreed in direction, and both favored the conventional sealer: 4 of 19 against 8 of 18 (RR 0.47, 0.17-1.30) [24] and 4 of 15 against 6 of 15 (0.67, 0.23-1.89) [56]. Heterogeneity was absent (I² = 0%, pQ = 0.645), the screen was passed, and the Mantel-Haenszel common-effect RR was 0.56 (0.27-1.15; p = 0.111; 67 teeth) (Figure 7). Against an assumed comparator risk of 424 complete resolutions per 1000 teeth, this corresponds to 189 fewer per 1000 (310 fewer to 62 more). The RE estimate was 0.56 (0.27-1.16) with τ² = 0. Leave-one-out omission left each individual estimate as reported above, and restricting to studies not at high risk of bias left the pooled estimate unchanged at 0.56 (0.27-1.15), because neither high-risk trial contributed to this outcome. The reverse fragility index was two events for one trial and four for the other, meaning the direction of the finding rests on very few teeth.

**Figure 7:**
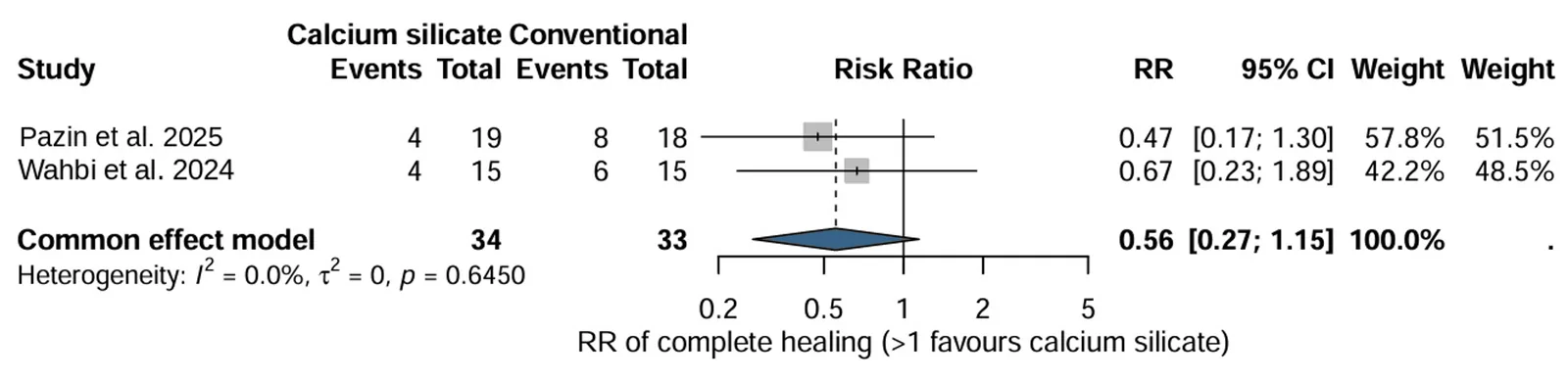
Periapical healing under strict criteria, randomized stratum Mantel-Haenszel common-effect RR 0.56 (95% CI 0.27–1.15), I² = 0% [24,56] RR: Risk ratio

In the non-randomized stratum, the single eligible cohort pointed the other way, with complete resolution in 19 of 28 against 8 of 16 (RR 1.36, 0.78-2.36) [21]. The design was confounded with imaging modality across these studies: both randomized trials used cone-beam computed tomography, and the only analyzable cohort used periapical radiography. Because three-dimensional imaging detects residual periapical rarefaction that two-dimensional radiography does not [34,61,62], the opposite directions may reflect the detection threshold of the modality rather than the design, and the two cannot be separated with the available studies.

Criterion Discordance

In the same 67 teeth, the point estimates pointed in different directions depending on the definition of success (Figure 8). Within each trial, this difference in direction was visible: 1.14 under loose criteria against 0.47 under strict criteria in one [24], and 0.93 against 0.67 in the other [56]. The post hoc ratio of RRs was 1.87 (0.89-3.90, p = 0.097) because the two estimates derive from overlapping patients and are positively correlated, treating them as independent overstates the variance, and this interval is conservative. Because the interval includes 1, the data do not establish that the effect differs by criterion; this analysis is exploratory and hypothesis-generating. A pattern in which teeth resolve at a similar overall rate but reach complete radiographic resolution less often by 12 months is consistent with a difference in the rate rather than the eventual fact of healing, but the present data cannot establish that interpretation.

**Figure 8:**
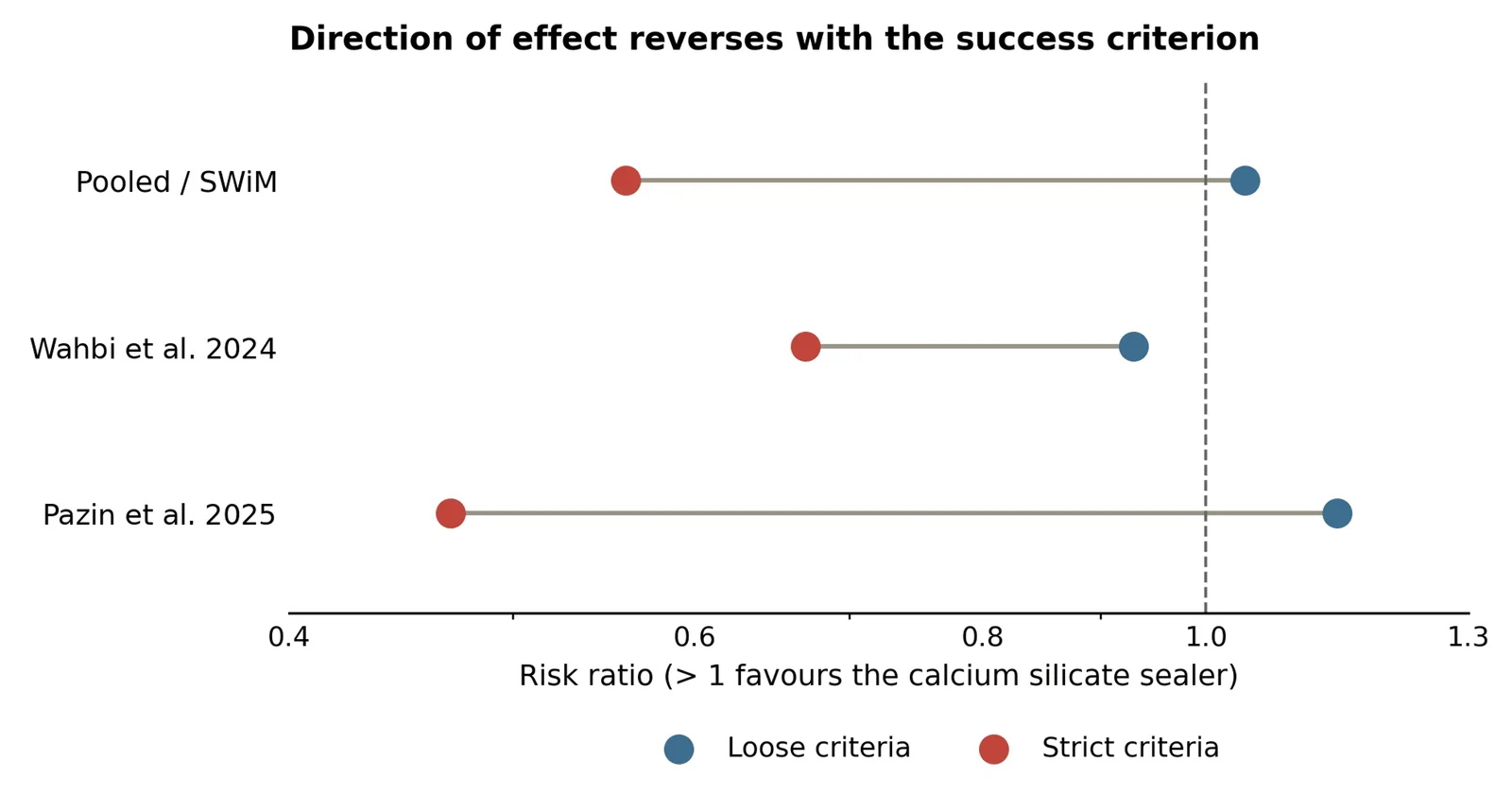
Direction of effect under loose and strict success criteria in the same teeth [24,56] SWiM: Synthesis Without Meta-analysis

Postoperative Pain, Analgesic Consumption and Sealer Extrusion

Two randomized trials contributed pain data against epoxy resin comparators at 24 and 48 hours (165 teeth) [54,55]. Heterogeneity was absent at both time points, and the screen was passed. The pooled RR for any pain was 0.93 (0.53-1.64) at 24 hours (Figure 9) and 0.85 (0.43-1.67) at 48 hours (Figure 10), corresponding to 16 fewer and 27 fewer episodes per 1000 teeth, respectively, with intervals spanning appreciable benefit and appreciable harm in both directions. Treating the split-mouth trial as parallel is conservative; assuming intracluster correlations of 0.2, 0.4, and 0.6 moved the 24-hour estimate only from 0.93 to 0.89.

**Figure 9:**
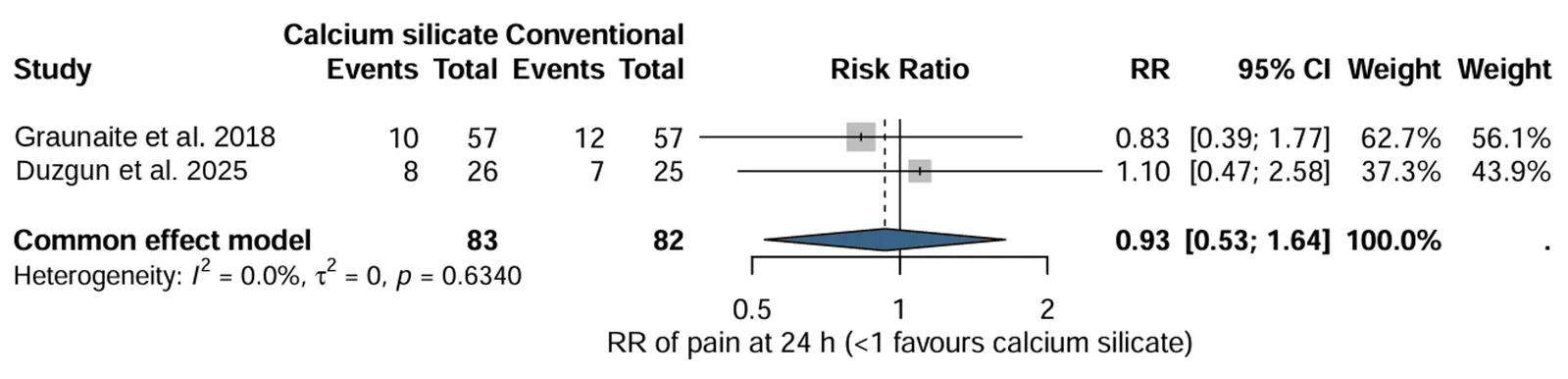
Incidence of postoperative pain at 24 hours versus epoxy resin comparators RR 0.93 (0.53–1.64), I² = 0% [54,55] RR: Risk ratio

**Figure 10:**
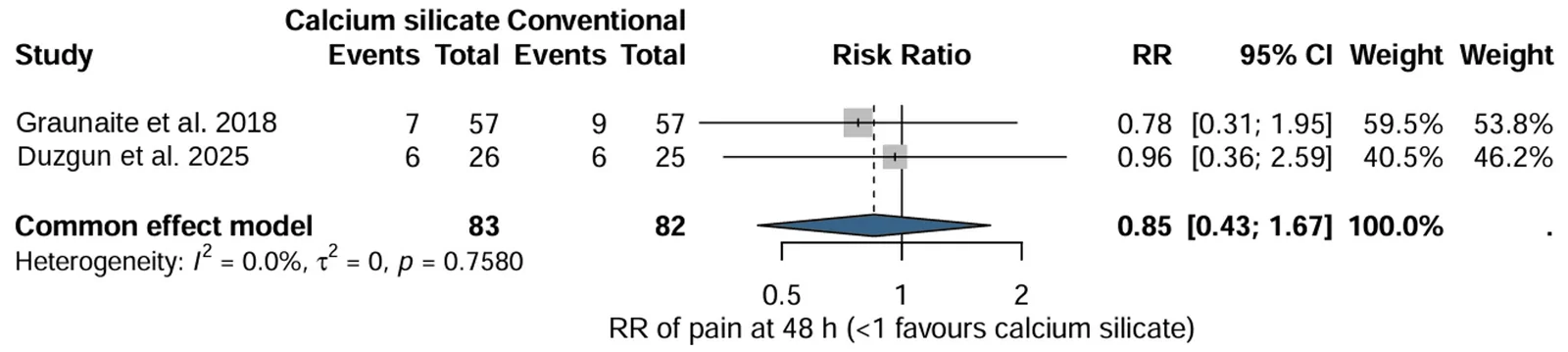
Incidence of postoperative pain at 48 hours versus epoxy resin comparators RR 0.85 (0.43–1.67), I² = 0% [54,55] RR: Risk ratio

All other pain time points rested on a single three-arm trial and were not pooled: 0.72 (0.37-1.41) at 6 hours, 0.96 (0.46-2.02) at 12 hours, 0.48 (0.17-1.40) at 72 hours, 0.48 (0.14-1.72) at 96 hours, 0.58 (0.15-2.16) at 120 hours, 0.64 (0.12-3.52) at 144 hours and 0.96 (0.15-6.31) at 168 hours [54]. Against the calcium hydroxide comparator in the same trial, the RR was 1.12 (0.49-2.54) at 24 hours and 1.12 (0.41-3.03) at 48 hours. Pain intensity was reported as a mean visual analogue score by one trial only, with a MD of -1.40 mm (-5.66 to 2.86) at 24 hours and −1.06 mm (−3.13 to 1.01) at 48 hours on a 100 mm scale [55]; the distributions are right-skewed, with means far below their standard deviations and a floor at zero, so these intervals should be read as optimistic.

Rescue analgesic consumption at 24 hours was reported by one trial, at 0.96 (0.27-3.43) [54]. A second trial recorded two analgesic users at 24 hours without stating allocation [55]; best-case and worst-case imputation bounded the estimate between 0.65 (0.20-2.11) and 1.45 (0.45-4.72), an interval that includes the null under every assumption. Apical sealer extrusion was reported in a retreatment-specific manner by one trial only, in which no extrusion occurred in either arm (0 of 57 and 0 of 57); with no events, no ratio measure is estimable, and the exact one-sided 97.5% upper bound on the true risk is 6.27% in each arm [55]. No study reported tooth survival separately for retreated teeth.

One non-randomized cohort reported a change in the Periapical Index as a continuous outcome, with a MD of 0.87 (0.13-1.61) favoring the calcium silicate sealer [21]. This outcome was reported as exploratory: the calcium silicate arm began from a substantially worse baseline, which mechanically enlarges the achievable reduction.

Subgroup and Co-intervention Analyses

Prespecified primary subgroup analysis could not be performed. The two studies in which the technique was matched, warm vertical compaction in both arms in one, lateral compaction in all three arms in the other, measured postoperative pain only [54,55]. Every study contributing healing data confounds sealer chemistry with obturation technique, with the single partial exception of the cohort in which both arms used a cold technique, though not an identical one [21]. Therefore, the matched level is empty rather than sparse. No other subgroup analysis reached the prespecified minimum of four studies per level.

Sensitivity to an Unresolved Numerical Inconsistency

One contributing trial reports figures for its comparator arm that are consistent with three different denominators: the stated failure rates of 5.3%, 16.7%, and 10.81% overall reconcile only at 18 of 19 and 15 of 18, whereas the reported tooth-type percentages imply 17 teeth and the stated loose success of 82.9% implies 34 of 41 [24]. The pooling decision is not stable across them: the 15 of 18 coding gives I² = 51.4% and fails the screen, the 15 of 17 coding gives I² = 19.9%, passes the screen and yields a common-effect RR of 1.005 (0.887-1.139), and the 34 of 41 coding gives I² = 69.0% and fails. The direction of the individual estimates is unaffected in all three, and the strict-criterion analysis is unaffected. The same report also states that 50 patients were randomized and 48 were evaluated at one year, which is incompatible with any of the three tooth-level denominators. The 19/18 coding was retained as primary because it reproduces the reported failure rates (1/19 = 5.3%, 3/18 = 16.7%, 4/37 = 10.81%) and complete-healing rates (4/19 = 21%, 8/18 = 44%).

Certainty of the Evidence and Distance from an Answer

Certainty was very low for every healing outcome and low for postoperative pain (Table 2). Healing outcomes were downgraded for risk of bias, for indirectness because sealer chemistry was confounded with obturation technique, and for imprecision; the loose-criterion outcome was downgraded for inconsistency. Pain outcomes were downgraded for risk of bias and for imprecision.

**Table 2:** GRADE summary of findings Randomized evidence started at high certainty, and non-randomized evidence at low certainty. Absolute effects are per 1,000 teeth and are constrained to the interval 0–1000. GRADE: Grading of Recommendations, Assessment, Development and Evaluation; RR: Risk ratio; CI: Confidence interval

Outcome	Stratum	k; teeth	Relative effect RR (95% CI)	Absolute effect per 1,000 teeth	Certainty	Downgraded for
Periapical healing, loose criteria (≥ 12 months)	Randomized	2; 67	Not pooled	Not estimated (heterogeneity screen not passed)	Very low	Risk of bias, inconsistency, indirectness, imprecision
Periapical healing, strict criteria (≥ 12 months)	Randomized	2; 67	0.56 (0.27–1.15)	189 fewer (310 fewer to 62 more)	Very low	Risk of bias, indirectness, imprecision
Periapical healing, loose criteria	Non-randomized	1; 44	Not pooled	Not estimated (single study)	Very low	Starts low; residual confounding, imprecision; indirectness (mean follow-up < 12 months in the calcium silicate arm)
Periapical healing, strict criteria	Non-randomized	1; 44	Not pooled	Not estimated (single study)	Very low	Starts low; residual confounding, outcome measurement, imprecision; indirectness (mean follow-up < 12 months in the calcium silicate arm)
Postoperative pain, 24 hours	Randomized	2; 165	0.93 (0.53–1.64)	16 fewer (109 fewer to 148 more)	Low	Risk of bias, imprecision
Postoperative pain, 48 hours	Randomized	2; 165	0.85 (0.43–1.67)	27 fewer (103 fewer to 123 more)	Low	Risk of bias, imprecision
Rescue analgesic, 24 hours	Randomized	1; 51	0.96 (0.27–3.43)	6 fewer (117 fewer to 389 more)	Very low	Risk of bias, imprecision
Apical sealer extrusion	Randomized	1; 114	Not estimable	No events in either arm (0/57 vs 0/57)	Very low	Imprecision
Tooth survival	-	0; 0	No data	No retreatment-specific data identified	No evidence	-

The imprecision judgements are quantified in Figure 11. Against the observed effect sizes, the evidence available represents 34.9% of the optimal information size for strict-criterion healing (67 of 192 teeth), 4.2% for loose-criterion healing (67 of 1,594), 2.8% for pain at 48 hours (165 of 5,856), and 0.8% for pain at 24 hours (165 of 20,712). No analysis approached the ten-study threshold prespecified for assessing small-study effects; therefore, funnel plots and Egger's test were not produced, and publication bias could not be evaluated.

**Figure 11:**
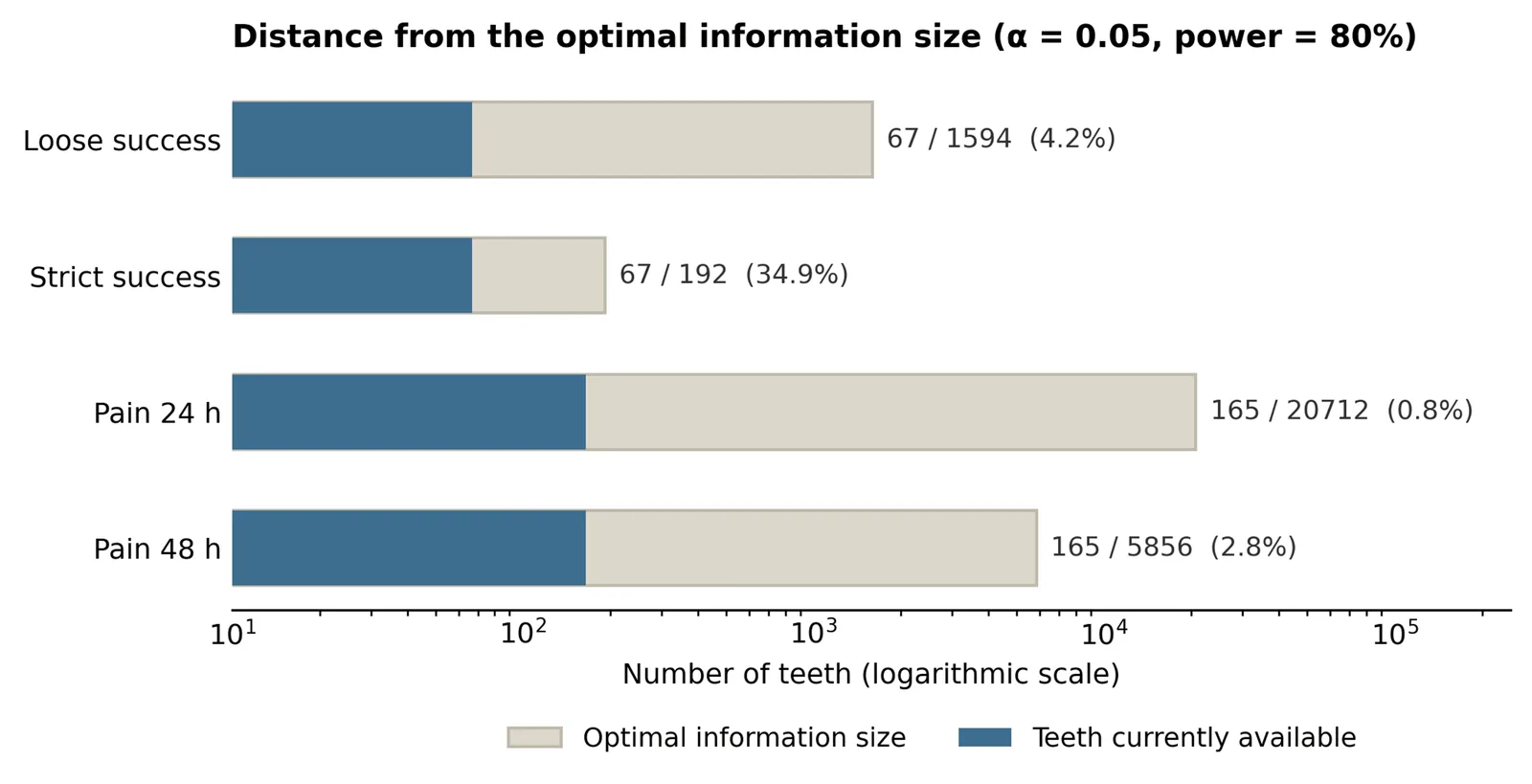
Teeth currently available against the optimal information size for each outcome, on a logarithmic scale (α = 0.05, power 80%) The strict-criterion healing outcome is the only endpoint within reach of a single feasible trial.

Discussion

This review restricts the question to nonsurgical retreatment and finds that the direction of the point estimate differed according to how success was defined. Under loose criteria, the two randomized trials disagree with one another and cannot be responsibly pooled. Under strict criteria, they agree, and the pooled point estimate favors the conventional sealer: 0.56, or 189 fewer complete resolutions per 1,000 teeth, with an interval that includes both a substantial disadvantage and a modest advantage for the calcium silicate sealer. This exploratory finding raises the possibility that the literature’s near-uniform conclusion of no difference partly reflects the definition chosen, although the ratio of RRs (1.87, 0.89-3.90) does not confirm a difference between criteria.

Loose criteria count a lesion that has shrunk alongside one that has disappeared; strict criteria count only the latter. A material that alters the rate at which a lesion resolves without altering whether it eventually resolves will therefore look identical under loose criteria at twelve months and different under strict criteria, and the difference will attenuate with longer follow-up. Both randomized trials assessed outcome by cone-beam computed tomography, which detects residual periapical rarefaction that periapical radiography misses [34,61,62], so the strict endpoint is more demanding and more sensitive than the endpoint most of the prior literature used. The single analyzable cohort, scored on periapical radiographs, pointed in the opposite direction under strict criteria.

The prespecified analysis of whether the obturation technique was matched between arms could not be run because no study contributing healing data matched it. In every such study, the calcium silicate arm was single-coned or sealer-delivered, while the comparator was warm-compacted or laterally condensed, and in one cohort, the correspondence was perfect [22]. These studies estimate the effect of an obturation strategy, not of sealer chemistry. This means that no published study can answer the question the field believes it is answering, and that a trial holding the obturation technique constant across arms would be more informative than another trial comparing the two strategies as packages. The two trials in this review that matched the technique measured pain only.

At the observed effect size, detecting a difference in postoperative pain at 24 hours with conventional power would require more than 20,000 teeth, explaining why five previous meta-analyses of sealer-related pain have returned the same null result [9,13-16] and why two umbrella reviews have consolidated it [18,19]: the cumulative literature is roughly 1% of the information required, so the repeated null is a statement about power, not about pharmacology. The contrast with the strict-criterion healing endpoint is instructive. That outcome is already at 35% of its optimal information size, and a single well-designed trial of approximately 192 retreated teeth would settle it.

Reviews of radiographic outcomes in mixed populations have reported no difference between sealer classes [7-12], as has the only previous review restricted to retreatment, which performed no quantitative synthesis [20]. Recent single-arm cohorts of calcium silicate-based retreatment report success at the upper end of the retreatment literature [25-27], which is compatible with our loose-criterion data and uninformative about the strict-criterion contrast because they have no comparator.

The principal strength is that the analytical decisions were fixed before the data were seen and were applied identically to the two co-primary definitions; therefore, the pooling decision could not follow the result. Where the prespecified screen withheld an estimate, the withheld estimate is nevertheless reported and labelled.

The limitations reflect the evidence rather than the method. First, the corresponding authors were not contacted for unpublished retreatment-specific data. Three otherwise eligible studies containing approximately 190 retreated teeth contributed to the narrative synthesis [57-60]. Furthermore, a major limitation is that the primary healing conclusions rely on only two RCTs comprising a total of just 67 teeth, which restricts the generalizability and robustness of these findings. A future update securing these data would bring the randomized healing stratum to three studies, at which point an RE model with a prediction interval becomes appropriate and would increase the pain evidence base by approximately 60%. Second, no outcome was informed by more than two studies. Therefore, between-study variance could not be estimated, and prediction intervals could not be produced; the reverse fragility indices of two and four events show how little separates the strict-criterion finding from a different one. Third, an unresolved numerical inconsistency in one trial determines whether the loose-criterion outcome is poolable, and all three codings are reported for this reason. Fourth, no analysis reached 10 studies. Therefore, small-study effects and publication bias could not be assessed. Fifth, the non-randomized evidence rests on a single cohort at serious risk of bias, in which the treating clinician scored the outcome unmasked, the arms differed substantially at baseline, and the mean follow-up of the calcium silicate arm fell below the 12-month threshold. Sixth, teeth rather than patients were the unit of analysis in most studies, and the clustering this induces is only partially addressed by the intracluster correlation sensitivity analysis.

Nothing in these data supports choosing between a calcium silicate-based and a conventional sealer for retreatment on the basis of periapical healing or early postoperative pain; the evidence is too imprecise to support a preference in either direction, and the point estimate under strict criteria is a reason for continued attention rather than for a change in practice. Three design features would materially advance the field: holding the obturation technique constant between arms so that sealer chemistry is isolated; reporting both loose and strict criteria for every outcome, with counts rather than percentages alone; and reporting retreated teeth separately whenever a mixed cohort is enrolled.

## Conclusions

In permanent teeth undergoing orthograde nonsurgical retreatment, the direction of the estimated effect of sealer chemistry on periapical healing differed with the definition of success, although the data do not establish that the effect depends on the criterion. Under loose criteria, the two available randomized trials disagree, and no pooled estimate is defensible; under strict criteria, they agree on a point estimate favoring the conventional sealer, at 0.56 (0.27-1.15), which the data are imprecise to confirm or exclude. Postoperative pain did not differ at 24 or 48 hours. Because sealer chemistry is confounded with obturation technique in every study reporting healing, the contrast measured is between obturation strategies rather than between sealers, and no causal claim about chemistry alone is supportable from the literature. Certainty is very low for healing and low for pain. A single trial of approximately 192 retreated teeth, assessed under strict criteria with the obturation technique held constant across arms, would be decisive.
